# Patient perspectives on the use of mobile apps to support heart failure management: A qualitative descriptive study

**DOI:** 10.1371/journal.pone.0285659

**Published:** 2023-05-11

**Authors:** Bridve Sivakumar, Manon Lemonde, Matthew Stein, Susanna Mak, Abdul Al-Hesayen, JoAnne Arcand

**Affiliations:** 1 Ontario Tech University, Faculty of Health Science, Oshawa, Canada; 2 Ontario Tech University, Social Research Centre, Oshawa, Canada; 3 Division of Cardiology, Department of Medicine, University of Toronto, Toronto, Canada; 4 Department of Medicine, Sinai Health, Toronto, Canada; 5 Unity Health, St. Michael’s Hospital, Toronto, Canada; Keele University, UNITED KINGDOM

## Abstract

**Background:**

Adherence to diet and medical therapies are key to improving heart failure (HF) outcomes; however, nonadherence is common. While mobile apps may be a promising way to support patients with adherence via education and monitoring, HF patient perspectives regarding the use of apps for HF management in unknown. This data is critical for these tools to be successfully developed, implemented, and adopted to optimize adherence and improve HF outcomes.

**Objective:**

To determine patients’ needs, motivations, and challenges on the use of mobile apps to support HF management.

**Methods:**

A qualitative descriptive study using focus groups (n = 4,60 minutes) was conducted among HF patients from outpatient HF clinics in Toronto, Canada. The Diffusion of Innovation theory informed a ten-question interview guide. Interview transcripts were independently coded by two researchers and analyzed using content analysis.

**Results:**

Nineteen HF patients (65 ± 10 yrs, 12 men) identified a total of four key themes related to the use of mobile apps. The theme ‘Factors impacting technology use by patients’ identified motivations and challenges to app use, including access to credible information, easy and accessible user-interface. Three themes described patients’ needs on the use of mobile apps to support HF management: 1) ‘Providing patient support through access to information and self-monitoring’, apps could provide education on HF-related content (e.g., diet, medication, symptoms); 2) ‘Facilitating connection and communication’, through information sharing with healthcare providers and connecting with other patients; 3) ‘Patient preferences’, app features such as reminders for medication, and visuals to show changes in HF symptoms were favoured.

**Conclusions:**

HF patients perceive several benefits and challenges to app use for HF self-management. Capitalizing on the benefits and addressing the challenges during the app development process may maximize adoption of such tools in this patient population.

## Introduction

Heart failure (HF) is one of the leading causes of morbidity and mortality worldwide, prevalent among approximately 64 million people globally [1]. Management of HF involves a combination of pharmacological treatments and/or device therapy, as well as modifications to diet and lifestyle (e.g., restriction to dietary sodium and fluid) [2]. A core component of HF management includes patient self-management, which involves monitoring and responding to changes in HF symptoms by titrating treatments, adapting behaviours and/or seeking care from a healthcare provider, as well as engaging in daily self-care activities (e.g., daily weighting) [3]. An integral part of HF self-care also includes adherence to medical and diet therapies. Adherence to such therapies has shown to reduce hospital readmissions and improve HF-related symptoms and survival rate [4, 5]. However, patient adherence to these therapies can be challenging, and high rates of nonadherence are common in this population, leading to decompensation and poor clinical outcomes [6–10].

Factors contributing to nonadherence among HF patients include lack of knowledge and misconceptions about HF management, lack of nutrition skills including difficulty reading food labels and identifying low-sodium products and lack of social support [11]. Moreover, HF patients are often burdened by multiple comorbidities, experiencing polypharmacy and complex treatment regimens, which can make treatment adherence and self-management difficult [12]. Traditional approaches to support self-management and adherence includes education via healthcare professionals, such as physicians, nurses, pharmacists, and dietitians, coupled with support from family and caregivers [13–16]. However, it can be difficult for some healthcare providers to assess and initiate these conversations in the clinical setting due limited time and subject matter expertise [17]. In addition, while patient education can increase knowledge about disease and self-management, education alone is not always sufficient to change behaviour. Therefore, interventions aimed to motivate and support behaviour change and maximize adherence are needed.

Mobile-Health (mHealth) technologies, defined as the use of mobile devices for healthcare, show promise in improving adherence. The use of such tools to support self-management has been encouraged by the American Heart Association [18]. Randomized controlled trials evaluating mHealth technologies for self-management and adherence in HF have demonstrated improved HF clinical outcomes including cardiovascular and all-cause mortality, New York Heart Association class, left ventricular ejection fraction, quality of life and physical functioning [19–22]. mHealth interventions have also been shown to improve daily symptom monitoring, dietary modification, medication adherence, physical activity, and self-efficacy, and support patients with multimorbidity [23–26]. mHealth technologies such as apps can support these positive health behaviours via education (content), self-monitoring, feedback, and gamification [27]. Overall, the evidence points to the strong potential for mHealth apps to support HF patients in self-management which involves treatment adherence.

A recent review identified 26 commercially available apps to support HF management, with the use of such apps increasing, even among older adults [28, 29]. However, when app quality is evaluated using generalized app evaluation tools (e.g., Mobile Application Rating Scale), only few apps developed for patients with HF scored highly for content, features, and functionality [30]. On further evaluation, many apps also lack features to support the acquisition of self-care knowledge and skills (e.g., engaging educational content, feedback), nor are they appropriate relative to the cognitive capabilities and technological literacy of patients with HF [28, 31]. In part, this may be due to the lack of research that has examined HF patient needs, challenges and motivations related to the use of mobile apps for HF management; research that has been conducted among healthcare providers, but not patients [32]. The importance of integrating end-user needs and values during intervention design has been emphasized in evidence-based models for behavioural intervention development, to optimize the relevance and impact of an intervention on the outcomes of interest [33]. Given the paucity of data related to mobile app use in HF, our study aims to determine patients’ needs, motivations, and challenges on the use of mobile apps to support HF management.

## Material and methods

A qualitative descriptive study using focus groups was conducted to explore patient perspectives on using mobile apps for HF management. This approach is rooted in naturalist inquiry and has been used in research informing development of health interventions [34]. The study was approved by the Research Ethics Board of Ontario Tech University (REB # 14882), the Unity Health Toronto Research Ethics Board (REB # 18–227), and the Mount Sinai Hospital Research Ethics Board (REB # 18-0212-E). Informed written consent was obtained from participants prior to interviews. Study reporting was guided by the consolidated criteria for reporting qualitative research (S1 Checklist) [35].

### Research team

The study investigators included a doctoral student (BS), two faculty members with experience in heart failure, behavioural intervention, and qualitative research methods (JA, ML), two HF cardiologists (SM, AA), and one social scientist with qualitative expertise (MS). There was no prior relationship between the interviewer (BS) and the participants.

### Study participants and recruitment

A purposive sampling strategy was used to recruit patients from two outpatient HF clinics in Toronto, Canada. Patients who were smartphone/tablet users aged ≥18 years with a diagnosis of HF were included. Eligible patients also had preserved ejection faction (>50%), mildly reduced ejection fraction (40–50%) or reduced ejection fraction (<40%) with the presence of HF symptoms (i.e., shortness of breath, fatigue or edema). Patients included were on stable medical therapy without hospital admission for > 6 months. Patients were excluded if they were unable to self-select food intake, unable to independently administer medications (i.e., living in a long-term care facility) and/or had difficulty communicating in the English language. Patients were identified and screened for eligibility by their treating physicians (SM and AA). Eligible patients were sent a recruitment letter inviting them to participate in the study. Interested participants met with the doctoral student during a clinic appointment. Informed consent was obtained either in person or via online consent form from patients prior to participation in the study. Participants were compensated $25 in the form of cash or gift card for their time.

### Data collection

Patients participated in focus group interviews (n = 4, 3–5 participants per focus group), led by a doctoral student (BS, female), with a research assistant recording observations about nonverbal cues. One-on-one interviews were conducted with patients who required accommodations. Qualitative studies usually require a minimum sample size of at least 12 participants; and three to six focus groups [36, 37]. Focus groups were chosen as the main method of data collection as they provide an opportunity to explore topics in-depth, allowing participants to build on each other’s ideas and insights [38]. Interviews were conducted in English and completed between June 2019 and March 2021. Interviews were conducted either on-site at the recruiting HF clinic or online via the Zoom platform (during the COVID-19 pandemic). Interviews were audio-recorded and lasted 45–60 minutes. Reflective notes were taken by the doctoral student after each focus group/one-on-one interview on the interview experience and unique perspectives shared by participants; and informed changes to wording and order of interview guide questions between subsequent focus groups. The decision to complete data collection was made by the research team when the intended number of focus groups (n = 3 to 6) was reached, and no new information emerged from the interviews.

A semi-structured interview guide (S1 File), consisting of ten open-ended questions and probes, was used to lead the focus groups/one-on-one interviews. The interview guide was developed by the research team and questions were informed by the five main constructs influencing the adoption of an innovation, described by the Diffusion of Innovation theory [39]. The five constructs include relative advantage, compatibility, complexity, trialability, and observability. All questions and probes were reviewed by the research team to ensure clarity and appropriateness. Participant sociodemographic and relevant medical information was collected from patient charts.

### Data analysis

The interviews were transcribed verbatim and anonymized, removing all identifiable information. The accuracy of verbatim transcripts was verified by a Research Assistant by comparing the transcripts to the audio-recordings. The computer software NVivo (version 12) was used for coding of transcripts and content analysis. Data analysis involved independent inductive coding of transcripts by two researchers (BS, MS), comparison of codes and sub-codes, establishment of intercoder agreement through collaborative discussion, followed by categorization of codes into themes. A theme reflected accounts of patient needs, motivations, or challenges on the use of mobile apps to support HF management. Themes were finalized through consensus from research team.

## Results

A total of 19 patients participated in the study and four focus groups were conducted. Two focus groups were held face-to-face and two were conducted online. Two individual one-on-one interviews were conducted (one face-to-face, one online). Participant characteristics are presented in Table 1. Overall, participants were 65.4 ± 10.2 years old and 12 were men. Most participants had reduced ejection fraction (n = 10) and the majority (n = 12) were New York Heart Association Class II. The reported frequency of technology use among participants included 0–2 hours/day (n = 4), 3–5 hours/day (n = 6), 5–8 hours/day (n = 4), and more than 10 hours/day (n = 4).

**Table 1:** Participant characteristics.

	Participants (n = 19)
Men (n)	12
Age (years)	65.4 ± 10.2
Living Arrangements	
Single/living alone	4
Married/living with spouse	9
Unknown	6
HF Diagnosis	
HF with reduced ejection fraction	10
HF with mid-range ejection fraction	4
HF with preserved ejection fraction	1
Unknown	4
NYHA Class	
Class I	6
Class II	12
Unknown	1
HF Duration (years)	
< 1	2
1–5	4
6–10	4
11–15	4
16–20	2
>20	3
Coronary Artery Disease	1
Hypertension	2
Diabetes	4
Dyslipidemia	5
Obstructive Sleep Apnea	6
Valve Disease	2
Smoking	2

### Patient usage of technology

Participants described using a variety of technologies, including laptops/desktop computers, mobile phones, and devices such as tablets for *communication and social media*, *daily activities* and for *monitoring their health*. Most participants stated that they used technology to communicate with their friends and families (e.g., phone, text, Zoom, email) and for social media. Several mentioned using apps for daily activities such as checking news, navigation, and weather, electronic banking, and shopping, and for entertainment and leisure purposes (e.g., movies, music, reading). Technology was also used by some participants to monitor their health, including tracking parameters such as daily step count and heartbeat.

### Patient views on adopting new technology

Many participants stated that they waited to get new technology and did not “feel a need to get new technology as it comes out”, some replacing their technology only when it was no longer working. They felt that if their current technology (e.g., phone, apps) “meets their needs” and is functioning well, they do not feel the need to “jump on the latest technology”. Prior to buying new technologies or downloading apps, they would read reviews, research its’ benefits, and rely on the recommendations of others. Certain participants identified themselves as “early adopters”, getting the latest technology as it comes out.

Four key themes were identified from the interviews: 1) Factors impacting technology use by patients, 2) Providing support through access to information and self-monitoring, 3) Facilitating connection and communication, and 4) Patient preferences of app features.

### Factors impacting technology use by patients

#### Opportunities

Participants identified several factors that can facilitate app use including having *credible information*, *having capabilities that are convenient and useful*, *an easy-to-use user-interface*, *opportunities for personalization*, and *opportunities to trial the app prior to adoption*, *and/or endorsement provided by their healthcare provider*. Several participants stated that they would use an app designed for HF management because it would be convenient and provide access to accurate information that is trustworthy. In addition to app content, some participants also felt that they would use an app for HF if it was *“useful”* for them and made their *“life better”*.

*“When I’m looking for information*, *I have to key in the information that I want to the site*, *read it through*, *and then again because it’s the internet*, *you can’t take that for truth*. *[…] Whereas if you have an app […] I don’t have to go search the internet for different things […] I have everything in one particular place*, *I know because the app is geared specifically to heart issues*, *I know that I can trust the information on the app*, *[…] here’s my information in an app*, *everything I might need or might require*, *it’s right here*, *given to me and it’s specifically geared to my issues […] that’s what would motivate me to use that app*”

[Individual Interview #2]

*“I guess*, *you know is this [app] going to be useful for me*? *You know if it’s not then I’m not going to download something just for the sake of it*”.

[Focus group #4]

Most participants emphasized that ease of use was an important factor for app use. This included a user-interface that is *“user-friendly*, *“intuitive”*, *“clear”*, and *“simple”*. Moreover, the information provided by the app should be short and easy to understand, not requiring a *“medical degree”*. Participants explained that if the interface was not easy to use it would be *“frustrating”*, *“confusing”*, and some even stated that it would be a *“deciding factor”* when it came to using an app. Some participants stated they would use an app for HF if they had the opportunity to trial the app prior to purchasing and/or if it was endorsed by their healthcare provider.

*“I believe in user-friendly*, *like if you give me an app and it’s like health related and I have to go through fifteen thousand menus…forget it*, *it’s such a turn-off*”.

[Focus group #1]

*“…yeah it would be when I sort of see a trial…because you don’t want to…if it’s an app that you have to purchase*, *for instance you don’t want to spend and find out it doesn’t do anything for what you’re looking for*”.

[Focus group #2]

Moreover, some participants identified that the ability to personalize an app was important for use. These participants felt that *“medical issues”* and *“need”* differ between individuals, and that patients can be better supported through personalization. For example, one participant commented:

*“Any program that is developed has to be able to expand not only from just heart failure but people who are diabetic*, *people of other type of medication requirements*. *It’s got to be all encompassing*, *otherwise I’m going to deal with maybe four or five types of apps in there to deal with each one of my illnesses*”

[Focus group #2]

#### Challenges

Potential factors impeding app use identified by participants included *being “inept” with technology*, *accessibility issues*, *privacy and trustworthiness*, *cost*, and *limitations with software/device capabilities*.

Few participants described themselves as being *“inept”* with technology, *“hardly using”* technological devices (e.g., computers, smartphones), and that they can become *“overwhelmed by all the apps”* on their cellphones. Several participants also identified that accessibility can be an issue for app use, especially for people with disabilities. Potential accessibility issues related to apps were typing, hearing, cognition, and memory (e.g., remembering various passwords, complexity of app interface), and sight (e.g., colour and layout of screen, need for bigger screens and space between letters).

Cost was also identified as a factor with participants stating that they would not want to pay for an app. Limitations with software/device capabilities were also cited as a barrier to app use by multiple participants. These limitations included the amount of space required by an app, *“glitches”* and *“crashes”* while using an app, and the *“dependence on having internet connection”*. Some participants stated they would worry about the privacy and trustworthiness of apps with personal information such as their email address.

*“I worry about the privacy of things…I signed up once for something […] I can’t remember what it’s called*, *but you know you share a little bit of information and suddenly they are emailing you all the time asking for more and you are like oh I wish I hadn’t shared that in the first place and unsubscribe*”.

[Focus group #2]

### Providing support through access to education and self-monitoring

Participants felt that an app for HF management could be supportive by *providing access to information about HF symptoms*, *prognosis*, *warning signs*, *diet*, *exercise*, and *medications*, as well as the *ability to monitor and track self-care activities*.

The majority of participants agreed that they could use an app as a source of reference for HF-related information. For example, several participants said that they *“don’t understand what heart failure is”*, or that *“technical”* terms such as ejection fraction is *“not well explained”*, therefore, such information could be provided on an app. Moreover, education on cardinal HF symptoms (e.g., tiredness, shortness of breath) and guidance on what to do in case of cardiac events (e.g., defibrillator shocks, arrhythmias, HF-related hospitalizations) were also stated to be useful. One participant said:

*“Well just as I said before*, *maybe some sort of you know reminder about a particular symptom that might occur that would require attention*. *I mean I’ve got a pacemaker; you know*, *I don’t know what might go wrong with a pacemaker*, *but you know if it started going down below a certain point for example*, *which never has*, *but I don’t even know if it could*, *you know*, *that kind of thing*. *And you know*, *I’ve got a couple of stents and I guess they do collapse from time to time*. *I don’t know what the symptoms would be if that happened*, *so maybe something along those lines*”.

[Focus group #4]

Several participants stated that access to diet-related information would be helpful in an app. This included having access to recipes and menus that are *“heart healthy”*, as well as information on healthier alternatives for foods and ingredients. One participant also saw value in having *“customized meal plans through an app”*. Moreover, guidance on what foods to consume versus what to avoid was also seen as helpful. This could be in the form of curated shopping lists, or information from a registered dietitian. Interestingly, some participants commented on the delivery of nutrition information, stating that most websites providing such information focus exclusively on cutting out foods that are *“bad for you”* (e.g., *“cut out all the sugars”*). These participants felt that this type of information was not useful, rather *“realistic”* advice and an explanation on why certain foods should be avoided or limited should be provided.

*“I mean I know salt’s a big one*, *I’m not going to look at every darn thing I’m eating here*, *you know what I mean I try to keep the salt low and […] doctors may have other suggestions about it*, *about doing it*, *that would be great in an app to know or to have a dietitian put some information into that app*”.

[Focus group #3]

*“[…] practical part of finding you know like cereal*, *you know I’ve experienced this but you like buying cereal in the store and trying to find low sodium and also low sugar content cereal is very*, *very difficult and…and so if…if when the dietitian or whoever said […] here are you know the top five in term of being low sodium and low sugar*, *and you know low fat or whatever and*, *and that would be a small thing but maybe quite helpful*”.

[Focus group #3]

Participants additionally emphasized that information on exercise in an app would also be helpful. This included information on exercises that are recommended for HF patients such as the amount of weight one should lift or types of exercises, as well as indicators to look for that signify that one should slow down or stop exercising. One participant stated:

*“I’ve had incidences in the past where I was doing what I thought was okay and then when I mentioned it to a doctor*, *they had said that is not the kind of exercise you should do and it’s too intense*, *but I had no idea*. *So*, *I think just that information*, *that have some limitations on what exercise we can and cannot do*, *it would be helpful to know that*”.

[Focus group #4]

Participants agreed that access to medication-related information on the app would be useful. Participants identified that they are on multiple medications for HF and other comorbidities; thus, it would be helpful to have information regarding medication interaction and potential side effects. For example, one participant shared an experience of taking a cold medication that interacted with their HF medications. Another participant expressed that they had *“multiple health issues”* and would like to know how the medications are *“affecting one another”*. Participants felt an app could be beneficial in providing an explanation of why medications are prescribed, guidance on what to do in case of missed doses, as well being able to store personalized medication information such as medication name, frequency, and dosage.

*“Again*, *for me the knowledge […] what are these pills doing*? *What are the side effects*? *You know*, *am I having this side effect because of this pill or because of something else I’m taking given the fact that I take so many things*, *right*? *Is it the heart issue*?”

[Individual Interview #2]

*“I always have to call in ‘hey [name of their doctor] I’m going to get a deviated septum surgery and the doctor prescribed me with steroid […]*, *can I take this with my heart medicine*?*’*. *So*, *to be able to do that on an app and say*, *‘will this interact*?*’ would be fantastic*”.

[Focus group #1]

Participants felt that an app can support monitoring and tracking of self-care activities. A few participants stated that a medication tracker may be useful as they sometimes miss doses due to forgetfulness and being on multiple pills. One participant shared that they currently used a spreadsheet and *“tick off”* medications as they take them, but would rather use an app, which would provide them *“assurance”* that they are taking their medication. Being able to record indicators such as blood pressure and weight was also viewed to be a helpful function in an app. One participant shared their experience using an app for tracking:

*“I use an app called [app name]*, *which is through one of my cardiologists that is again just sort of tracking day-to-day where you’re at and then they will call me if there is any changes or if they have questions*.”

[Focus group #4]

### Facilitating connection and communication

It was viewed that apps can potentially help patients *connect and share information with their healthcare providers*, *connect and talk with other patients*, and provide a medium *for caregivers to connect and communicate*.

Most participants saw an opportunity for apps to make information sharing easily accessible for both them and their healthcare provider. Many also expressed that they often feel a *“sense of isolation”* and *“alone”* in managing their HF; and if data such as their blood pressure readings, symptoms, medications, test results can be shared with their healthcare provider through an app it would provide them with a *“sense of encouragement that you’re connected in some way”*. They felt that this information can help healthcare providers keep track of their health and help flag problems. Some participants also said that they occasionally feel unwell in between clinic appointments, however they forget to recount these episodes with their healthcare provider. If they were able to capture these periods of time on an app as it happened, they could easily share this information with their healthcare provider.

*“…you can take everything with you when you go*, *your information*, *your medication list you know*, *you can log all your symptoms…you know the names are so difficult*, *so you just put it there and you don’t have to talk just show it to the doctor*”.

[Focus group #2]

*“It would be especially good if it’s information that the cardiologist would be interested in*. *I don’t mean that the cardiologist should check in on you every day*, *but you know when I next come for an appointment*, *he can look at the app and see what my blood pressure’s been over the last six months and whether or not I’ve been exercising or whatever the information that would be useful to the doctor in monitoring my progress or lack of would be good*”.

[Focus group #4]

Several participants saw value in connecting with other patients through an app. They felt that an app has the potential to facilitate social support, a sense of community, and support their mental health.

*“…helps to talk to people who are going through the same stuff because family they don’t always understand*”.

[Focus group #1]

*“I would have a community*. *And maybe if I start to get symptoms and I want to talk to somebody about them*. *It might be interesting to just say ‘have you had this*? *And what happened*?*’*”.

[Individual Interview #1]

Multiple participants expressed that this type of support system through an app may be especially beneficial to newly diagnosed patients. In contrast, a few participants expressed that they do not a feel a need to connect with other HF patients. Interestingly, one participant felt that it would be of value to offer a medium for caregivers to connect with each other:

*“The person that really needs to come together with other people is the caregiver…they are the ones that have to really understand what we are going through […] they don’t know what you are experiencing and how that experience translates into something else or what they can do to be able to improve your circumstance…my wife is my caregiver…she is the one that reads all the labels…nothing gets past her in terms of sodium…she knows exactly how much sodium is in everything that I put in my mouth […] but these are the people that really need to communicate with other people*”.

[Focus group #2]

### Patient preferences of app features

Participants shared their views on potential app features to be integrated into an app for HF management including *alarms or reminders for medication*, *exercise and appointments*, *goal setting features*, *connectivity between other apps and devices*, *graphs*, *and other visuals to show progress and changes in symptom indicators*, and *gamification*.

Several participants felt that it would be useful for an app to include alarms or reminders for HF self-care activities such as taking medications, tracking blood pressure and weight, as well as exercise. A few participants also suggested that being able to set goals on an app such as step counts can be *“motivating”* and *“encouraging”*. Moreover, visual representations such as graphs could be used to show progress on goals and track symptoms over time.

*“It’s sort of that visual representation that I actually accomplished my goal of ten thousand steps for the week and I just say that because you know it’s something that they use on the Peloton app*, *the Apple app*, *and things that have been very successful so I think for some people that can be a motivating factor*”.

[Focus group #4]

*“My background is research*, *I like graphs*, *I think graphs and you know spreadsheets are very informative and I will create a graph for my doctor and show him my blood pressure changes over last ten years*, *and that would have made an enormous difference in terms of her decision making*”.

[Focus group #3]

The ability to have health information connected across apps and devices was also viewed as a helpful app feature, as highlighted by one participant:

*“[…] you know I use the Apple health app for example*, *and I love the ability to connect different things*. *So*, *if there’s like an exercise app where it’s tracking your heart rate and all of this be linked together*, *so we would have like an accurate portrayal of our health at any given time*, *so it’s related to weight*, *your medications*, *etc*.”

[Focus group #4]

There were mixed views on a gamified app for HF. Some participants expressed that they *“have never taken to games”*, they would find it *“annoying”*, that it would be a *“good idea for younger people”*. A participant also questioned the longevity of a game, stating that it would be *“interesting for the first little while”* but that people might not return to it. One participant mentioned that gamification with the intent of being educational could be acceptable, but with a caveat, stating:

*“don’t treat us as if we are in kindergarten*, *you know there’s that thing about older people*, *you know you here it especially when they get into cognitive impairments*, *you know they are just like kids*, *no they are not just like kids*”.

[Focus group #3]

Conversely, few participants stated that games, such as those focused on cognitive skills could keep the *“mind young and active”* and that gamification elements such as badges for reaching achievements could be *“motivation for some people”*.

## Discussion

To our knowledge, this is one of the first studies to explore HF patients’ perspectives on the use of mobile apps for HF management, highlighting their technology and app use behaviours as well as several opportunities and challenges. These data are highly relevant considering the emergence of many HF management apps, and the scarcity of data on patient perspectives related to these apps. Participants identified factors impacting app use (e.g., accuracy of information, accessibility), and ways in which apps can be supportive, such as providing access to information and self-monitoring and facilitating connection and communication. App features preferred by patients including alarms and reminders and goal setting were also identified. These findings demonstrate the potential for mHealth apps to be an acceptable tool for supporting patients with self-management of HF.

A common view is that HF patients may be unwilling to use modern mHealth technologies such as an app for HF management due to old age and potential lack of technological literacy [40, 41]. Our findings suggest the opposite. Participants of our study reported using a variety of technologies for aspects of living including monitoring of their health, demonstrating that technology has become well integrated into their everyday life. This finding is consistent with studies like Leigh et al. [42] who found that 68% of patients with HF owned smartphones and 53% expressed interest and willingness to use mHealth apps for HF self-care. Similar findings were also observed among older adults with HF [43]. However, adoption of apps among HF patients may be short-lived [31], which emphasizes the importance of exploring patient perceived barriers and facilitators of app adoption.

This study found that accessibility issues such as typing, screen size, cognition and memory, and hearing were a potential challenge to app adoption. This finding is supported by a review that identified usability features in mobile devices that found that device size and the complexity of interfaces are barriers to mHealth adoption [44]. Since most patients with HF are older adults who may experience age-associated physical and cognitive decline, integrating accessibility features such as digital zoom, speech-to-text, audio, and the conservative use of colours is critical to support app use among older adults [44, 45]. Evidence suggests that a technology that is intuitive will be more readily accepted as it requires less time and mental effort to learn and operate [44, 46]. This was echoed by our participants who shared that they preferred an app interface that was simple, user-friendly, intuitive, and simple. The various barriers and facilitators to mHealth adoption identified in the literature and in our study reveal the importance of involving patients in the design and development of mHealth apps. Although patients may not have technical knowledge, they can provide valuable insights into their needs and capabilities which can yield better app uptake, especially among those that may be less technology savvy [43]. As recommended by authors such as Davidson et al. [47] and Cajita et al. [43], the findings of this study highlight the value of the participatory approach in developing mobile apps for HF management to address patient needs and maximize usability, accessibility and adoption in this population.

Our study identified several patient-reported opportunities for supporting HF management, including medication and diet adherence, via mobile apps. Examples of these opportunities include being able to identify medication side effects or healthier alternatives to foods and ingredients that are high in sodium, as well as knowing what to do when one misses a medication dose and what foods to consume versus the foods to avoid. In contrast, three notable reviews found that the majority of HF management apps primarily focused on symptom monitoring, with a few apps including features focused on diet and medication [28, 31, 48]. While monitoring and tracking are important components of HF self-management, alone they are insufficient to engage patients in adhering to self-care recommendations. HF patients require certain knowledge and skills to support effective behavioural actions and high levels of self-efficacy in HF management. In addition to our data and published systematic reviews, HF healthcare providers have expressed support for mobile apps for HF-related patient education [32]. Indeed, the opportunity for using mHealth to support diet and medication adherence in HF aligns with empirical evidence demonstrating that mHealth interventions that incorporate education and e-counselling can improve self-care related to cardiovascular disease risk factors [49, 50]. Therefore, alongside monitoring, app developers should integrate engaging educational elements into their apps to support patients in having the knowledge and skills to successfully execute and sustain behaviour changes related to the medication and dietary regimens.

Participants in this study also shared the potential for mobile apps to be a medium for information-sharing and for facilitating connection and communication with healthcare providers. These are opportunities also identified by HF cardiologist and nurses [32]. Although connection and communication through mobile apps between patient and healthcare provider can support information gathering and clinical decision-making, its practical implementation in clinical practice must addressed. Clinic staff support may be required to train patients on how to use such app functions [51]. The amount of electronic data, clinician workload, and administrative work produced by mHealth tools can impede their adoption in clinical practice [32, 52, 53]. Thus, adequate infrastructure, education/training for patients as well as healthcare providers on using digital health technologies, and integration protocols for these tools in clinical practice are needed to support the functionality and success of digital health tools in the clinical setting [52, 54]. Moreover, involving healthcare providers in the design and development of mobile apps can also be helpful to support the successful implementation of these tools in the clinical setting.

An interesting finding from our study warranting discussion is participant experience of multimorbidity, which was a cross-cutting theme. Participants identified opportunities for HF mobile apps through their experience of comorbidities, including providing education on medication interaction and side effects, as well as monitoring and tracking self-care activities. Research exploring impact of mHealth apps on multimorbidity self-management support are limited; most studies focus largely on a single disease population [55]. One review identified that existing health apps for patients with multimorbidity are of acceptable quality; however, the authors concluded that the apps have limited potential in promoting behaviour change [26]. Moreover, to our knowledge only one app has been designed and validated to improve health literacy and self-management among patients with multimorbidity and HF [56]. Due to factors such as disease burden, polypharmacy, and complex treatment regimens, individuals with multimorbidity will often need personalized and multi-faceted self-management support [12, 57]. Thus, further research is needed to explore the development, implementation, and effectiveness of mHealth apps in supporting patients with multimorbidity and HF.

Participants in this study desired app features including medication reminders, goal setting, sharing of information across devices, and graphs, visuals to show trends in HF symptoms and progress, as identified in previous research for management of chronic diseases such as diabetes [58]. Our study uniquely found that HF patients’ views on gamification were mixed. Gamification is the highly common and popular use of gaming elements and techniques [e.g., goal setting, reinforcements], in a non-game context, to drive behavioural change. Gamification in the context of HF management is new and is distinctly different from serious games (i.e., video games designed for the purpose of education). mHealth apps can include gamification elements without being a game itself. Results of our study suggests that HF patients may not prefer serious games, but rather the integration of gamification elements (e.g., points, badges) into an HF app. Evidence suggests that such elements can be effective in improving self-efficacy, motivation, medication adherence, knowledge, and engagement in exercise training; and acceptable for supporting self-management among patients with cardiovascular disease [59–62]. Therefore, gamification may offer a potential mechanism for improving adherence and self-management among the HF population and further research in this area would be worthwhile.

Our study has limitations that must be discussed. Firstly, a part of our data collection period coincided with the COVID-19 pandemic resulting in interviews being conducted both in-person (2 focus groups, 1 individual interview) and online (2 focus groups, 1 individual interview). Although online focus groups and interviews are comparable to in-person, we acknowledge that some limitations such as minimal interpersonal contact and interaction exist [63, 64]. However, equal number of interviews were conducted in-person and online and participants in the online interviews were all on video where non-verbal cues could be assessed, closely resembling in-person interviews. Secondly, individual interviews were conducted to accommodate some participants, which can differ in methods compared to focus groups (e.g., group interactions). However, we anticipate the impact of these differences are minimal given only two individual interviews were conducted, with the analytic approach remaining consistent (inductive coding, content analysis). Thirdly, we had slightly more men than women participants, which is relevant since there may be gender differences related to HF self-care and app use [65, 66]. Additionally, perspectives shared by participants in this study are limited to HF patients that are smartphone/tablet users, clinically stable and English speakers. Lastly, the study findings may have inherently been influenced by the beliefs and assumptions of research team members. To acknowledge these influences, we employed reflexive strategies such as reflective memoing by the interviewer. Moreover, participants quotes are provided to support findings and interpretations.

## Conclusions

HF patients perceived several benefits and challenges, as well as opportunities for app use for HF self-management. Capitalizing on the opportunities that apps offer, and addressing patient perceived challenges, during the app intervention design and development process may better support the adoption of such mHealth tools in this patient population. This may be achieved by actively engaging patients in the conceptualization, design, development, iterative testing, and evaluation of apps through a participatory approach. This approach will allow researchers to ensure apps are designed to meet the unique needs and capabilities of HF patients.

## Supporting information

S1 File Interview guide. (PDF)

S1 Checklist COREQ checklist. (PDF)
