# Supplementary material for: Patient perspectives on the use of mobile apps to support heart failure management: A qualitative descriptive study
Source: PLoS One. 2023 May 11;18(5):e0285659. doi: 10.1371/journal.pone.0285659 (PMC10174481; doi:10.1371/journal.pone.0285659)
Supplement: S1 File — (PDF) [file pone.0285659.s001.pdf]

Interview Guide

Thank you for joining us for today's 45-minute focus group/interview. The goal of this interview is to get an understanding of your views on using technology, like mobile apps for managing heart failure. With your permission, may we audio-record this interview? If you have not yet reviewed or completed the consent form, there is time to do so now. If you have any concerns and/or questions, please feel free to ask us. We would like to remind you that participation in this study is entirely optional, and you may leave the study at any time without it affecting your care. You may refuse to answer any question you do not want to answer, by saying "pass". The information that you share will be kept confidential and will not be shared with anyone outside of the study. Before we begin, if you have any questions or concerns throughout the interview, please feel free to ask.

Adoptive Characteristic/Complexity

Q1) How comfortable *(feeling of ease)* are you with using your smartphones/tablets?

a. 'Comfortable': For example, do you use it only when you have to (like to make a call or answer a text) or do you find yourself on there when you have down time to play around (like to use social media or checking emails)
b. What do you find difficult about using smartphones/tablets?
c. What do you find easy about using the smartphones/tablets?
d. Do you find yourself downloading apps?
    i. If yes, what type of apps? (e.g. games, lifestyle, email, social media)
    ii. If no, why not?

Q2) Describe yourself when a new technology comes on the market?

a. Do you generally try to always get the newest device (eg. Phone or tablet model) or latest application?
b. Do you wait until a technology has been on the market for some time to ensure it is worth using?
c. Do you usually rely on your work or kids to keep you up to date with technology?
d. Do you wait until you absolutely have to make a switch or embrace a new technology?

Q3) What do you look for when selecting an app?

a. Are you looking at the media that the app uses (i.e text, video, games, audio) or are you more concerned with the content *(information)* that the app provides?
b. Do you feel that an app that is catered towards your needs is important? If yes/no Why? *(Example: an app that allows you to input your health information, based on which info regarding diet and medication is provided)*
c. Is aesthetics *(the look of the app)* important to you? If so, how?
d. Would you look for the same elements when you are using an app for diet and medication management?

Q4) If you could design your own app to help you manage heart failure, what are some of the content areas *(information)* that you would want your app to focus on?

Q5) If you could design your own app to help manage heart failure, what are some motivational elements *(features that can help you)* you would want the app to have?

Q6) Do you see value *(helpfulness, easiness)* in connecting with other individuals effected by heart failure?

a. Do you find it hard to find people?
b. What if there was an online option to hear others' stories or share tips on managing heart failure, would that be something you would be interested in using?

Relative Advantage

Q7) How do you feel about using technologies, such as mobile apps to manage heart failure, specifically with helping you follow your dietary and medication instructions?

a. How do you think it will be advantageous *(helpful)?* Or disadvantageous *(not helpful)*?

Q8) Presently, do you use any health and/or lifestyle apps to manage heart failure?

a. If yes, what app do you use?
b. How do you use this app to manage heart failure?
c. How does this app meet your health goals and needs?
d. What do you like about the app?
e. What do you dislike about the app?

Compatibility

Q9) How can an app for heart failure management be compatible *(fit-in; work well with)* with your health needs, goals, and lifestyle?

a. What part of managing heart failure do you find the most challenging? (e.g., polypharmacy)

Trialability/Observability

Q10) From your perspective, what would make you use an app for heart failure management?
