# Supplementary material for: Patient perspectives on the use of mobile apps to support heart failure management: A qualitative descriptive study
Source: PLoS One. 2023 May 11;18(5):e0285659. doi: 10.1371/journal.pone.0285659 (PMC10174481; doi:10.1371/journal.pone.0285659)
Supplement: S1 Checklist — (PDF) [file pone.0285659.s002.pdf]

Patient perspectives on the use of mobile apps to support heart failure management: A qualitative descriptive study

Consolidated criteria for reporting qualitative studies (COREQ): 32-item checklist

No. Item	Guide questions/description	Reported on Page #
**Domain 1: Research team and reflexivity**		
Personal Characteristics		
1. Interviewer/facilitator	Which author/s conducted the interview or focus group?	Material and methods: Research team, Data collection, p.5-6
2. Credentials	What were the researcher's credentials? E.g. PhD, MD	Materials and methods: Research team, p.6
3. Occupation	What was their occupation at the time of the study?	Material and methods: Research team, p.6
4. Gender	Was the researcher male or female?	Material and methods: Data collection, p.7
5. Experience and training	What experience or training did the researcher have?	Material and methods: Research team, Data collection, p.6
Relationship with participants		
6. Relationship established	Was a relationship established prior to study commencement?	Material and methods, p.6
7. Participant knowledge of the interviewer	What did the participants know about the researcher? e.g. personal goals, reasons for doing the research	Material and methods: Research team, p.6; S1 Interview Guide
8. Interviewer characteristics	What characteristics were reported about the interviewer/facilitator? e.g. Bias, assumptions, reasons and interests in the research topic	Material and methods: Research team, p.6; S1 Interview Guide

**Domain 2: study design**		
Theoretical framework		
9. Methodological orientation and Theory	What methodological orientation was stated to underpin the study? e.g. grounded theory, discourse analysis, ethnography, phenomenology, content analysis	Material and methods, p.6
Participant selection		
10. Sampling	How were participants selected? e.g. purposive, convenience, consecutive, snowball	Material and methods: Study participants and recruitment, p.6
11. Method of approach	How were participants approached? e.g. face-to-face, telephone, mail, email	Material and methods: Study participants and recruitment, p.7
12. Sample size	How many participants were in the study?	Results, p.9
13. Non-participation	How many people refused to participate or dropped out? Reasons?	N/A
Setting		
14. Setting of data collection	Where was the data collected? e.g. home, clinic, workplace	Material and methods: Data collection, p.7 .
15. Presence of non-participants	Was anyone else present besides the participants and researchers?	Material and methods: Data collection, p.7
16. Description of sample	What are the important characteristics of the sample? e.g. demographic data, date	Results, p.9; Table 1
Data collection		
17. Interview guide	Were questions, prompts, guides provided by the authors? Was it pilot tested?	Material and methods: Data collection, p7-8; S1 Interview Guide
18. Repeat interviews	Were repeat interviews carried out? If yes, how many?	No, inferred in Methods: Data collection, p.7

19. Audio/visual recording	Did the research use audio or visual recording to collect the data?	Material and methods: Data collection, p.7
20. Field notes	Were field notes made during and/or after the inter view or focus group?	Material and methods: Data collection, p.7
21. Duration	What was the duration of the interviews or focus group?	Material and methods: Data collection, p.7
22. Data saturation	Was data saturation discussed?	Material and methods: Data collection, p.7-8
23. Transcripts returned	Were transcripts returned to participants for comment and/or correction?	N/A
**Domain 3: analysis and findings**		
Data analysis		
24. Number of data coders	How many data coders coded the data?	Material and methods: Data analysis, p.8
25. Description of the coding tree	Did authors provide a description of the coding tree?	N/A
26. Derivation of themes	Were themes identified in advance or derived from the data?	Material and methods: Data analysis, p.8
27. Software	What software, if applicable, was used to manage the data?	Material and methods: Data analysis, p.8
28. Participant checking	Did participants provide feedback on the findings?	N/A
Reporting		
29. Quotations presented	Were participant quotations presented to illustrate the themes/findings? Was each quotation identified? e.g. participant number	Results, p 12-22 (Yes)
30. Data and findings consistent	Was there consistency between the data presented and the findings?	Results, p 12-22 (Yes)
31. Clarity of major themes	Were major themes clearly presented in the findings?	Results, p 12-22 (Yes)
32. Clarity of minor themes	Is there a description of diverse cases or discussion of minor themes?	Results, p 12-22 (Yes)
